# A narrative review of Master’s programs in midwifery across selected OECD countries: Organizational aspects, competence goals and learning outcomes

**DOI:** 10.18332/ejm/188195

**Published:** 2024-06-13

**Authors:** Angela Kranz, Anja A. Schulz, Konstanze Weinert, Harald Abele, Markus Antonious Wirtz

**Affiliations:** Section of Midwifery Science, Institute of Health Sciences, University of Tübingen, Tübingen, Germany; Research Methods in the Health Sciences, University of Education Freiburg, Freiburg, Germany; Department for Women’s Health, University Hospital Tübingen, Tübingen, Germany

**Keywords:** higher education, narrative review, learning outcomes, postgraduate education, Master’s programs in midwifery, competence goals

## Abstract

Shifting midwifery education to a university level is of great importance for healthcare systems worldwide by preparing graduates for current and future challenges. Some of them referring to management, research and teaching tasks as well as advanced practitioner roles, require competences that can only be acquired in a Master's program. The objectives of this narrative review are to outline the differences and commonalities of organizational aspects of Master’s programs in selected OECD countries and to point out the competence goals and learning outcomes they are based on. Fifteen Master’s programs in twelve OECD countries were identified and analyzed. Considering the organizational characteristics, differences are found in admission requirements and qualification levels, while similarities relate to the awarded title (MSc). All programs aim to develop abilities for research to advance midwifery practice. Leadership and management abilities are addressed through effective teamwork and communication. The programs’ aims are to develop abilities for midwifery education tasks. Whereas competence goals mostly align across the programs, they are addressed differently through various learning outcomes. Development and enhancement of Master’s programs in midwifery are needed by focusing on core elements, such as common competence goals. It is equally important to adapt them to national healthcare and educational systems.

## INTRODUCTION

Academization of the healthcare professions is of great importance for healthcare systems worldwide^1^. Not only should the professions be made more attractive^1^, but graduates should also be prepared for the increased future challenges of their professions^1,2^. This development is reflected within in the midwifery profession, as it became more complex in recent decades due to a variety of factors^3,4^. These include epidemiological trends such as the increasing age of first-time mothers^4,5^ as well as socio-economic challenges, like the growing number of women with a migration background^4,6^. Political and economic factors include cost-efficient and nationwide healthcare^4,7^, whereas for example clinical challenges arise such as higher rate of multiple birth due to fertility treatments^4,6^. As a result, midwives are faced with increased demands on their working environment and practice^4^. The huge midwifery shortage worldwide reinforces these developments^4,8^. To deal with these challenges, there is a need for well-educated and competent midwives^3,8^. In recent decades, midwifery education shifted to a university level^8-10^, mostly to ensure autonomy and professionalization of the midwifery profession^11,12^. Many of the countries in the World Health Organization (WHO) regions allow primary midwifery qualification through a three- or four-year Bachelor’s program^9,13^. As international standards for midwifery education do not specify the minimum level of qualification^14-16^, there are different pathways for pre-service education for midwives worldwide [direct entry programs (e.g. Bachelor’s degree), post-nursing programs and integrated programs (nursing and midwifery qualifications combined)^9^]. Bachelor’s programs for primary qualification are often seen as a suitable way to react to the shortage of midwives as well as to provide appropriate skills and abilities for new starters^9,17^. But it is known that the minimum level and quality of midwifery education often does not adequately prepare midwives for their professional practice^13^. There are challenges that cannot be met with a Bachelor’s degree, especially referring to management^13,14,18^, research^19^ and teaching^20,21^ tasks as well as for advanced practitioner roles^22^.

Midwives with excellent management skills are needed to meet the aforementioned challenges^21,23^. The International Confederation of Midwives (ICM) recommends the following principles to ensure that midwifery management is effective: inspire, influence, advocate, collaborate, communicate, challenge the status-quo, be accountable, and demonstrate compassion^14,18^. Through Master’s programs, midwives can further develop the relevant skills recommended by ICM^14,18^. They educate skills far beyond Bachelor’s level not only in management, but also advanced pedagogical skills to enable midwives to undertake educational tasks^20,21^. Beyond that, research skills are deepened during Master’s programs^19^. All these taught areas allow midwives to meet the mentioned challenges by expanding and re-organizing education, personnel planning, ensuring a positive working environment and innovative evidence-based practice^21,23^, as well as further develop the midwifery profession^24^.

### Status quo of Master’s programs in midwifery

As more countries worldwide develop Bachelor’s programs for midwives^8,25,26^, it is essential to create opportunities for further academic qualification^10^. Compared to Bachelor’s programs, considerably fewer Master’s programs in midwifery are implemented^8,25^ although it is recommended that midwifery education should be offered at Bachelor’s and Master’s level^26,27^. Internationally, barely a quarter (26%) of the 74 included countries in the State of the World’s Midwifery (SoWMy) report indicate that a Master’s program is the highest qualification, while another quarter (27%) offer a doctorate for midwives^8^, mostly in high-income countries^8^. In Europe, all Bachelor’s, Master’s and doctoral programs are described and standardized by the European Qualification Framework (EQF)^28,29^. To use these levels internationally, the International Standard Classification of Education (ISCED)^[Bibr cit0030]30^ is used. Both EQF^28^ and the ISCED^30^ describe the Master’s degree at level 7. Programs at level 7 aim to enable participants for advanced academic and/or professional knowledge, skills and competencies^30^. Despite these general standardizations, there are no further standards for Master’s programs in midwifery, as most standards apply for primary qualification of midwives^14-16^. While some Master’s programs serve as primary qualification through post-nursing programs^9^, most of the Master’s programs in midwifery are designed as postgraduate qualification after a Bachelor’s degree^9^. The international lack of standardization or recommendations for Master’s programs in midwifery, leads to the fact that they differ at national, European as well as international level, especially in terms of duration, content and results^10,12,23,26^. Nevertheless, studies show a trend of acquired competences in Master’s programs. Midwives with a Master’s degree have a higher level of pedagogical, management and research skills^21^, which is why these areas will be outlined in the following.

### Master’s programs in midwifery: Leadership and management

The midwifery profession is traditionally predominantly practiced by women worldwide. To strengthen midwifery and to motivate and retain qualified professionals, the establishment of midwifery leadership opportunities are crucial. Still, challenges remain at both policy and clinical levels^13^. A lack of female leaders in the healthcare systems, as well as limited leadership opportunities for midwives often results in midwives not climbing the leadership ladder. At the same time, decisions about midwifery profession are often not made by leading midwives themselves. Especially, if midwifery is incorporated into the structures of nursing, midwives have limited leadership opportunities^8^. According to the SoWMy, 71% of the reporting countries state that at least one midwife holds a leadership position^8^. Nevertheless, midwives need to be supported when it comes to practicing their role as leaders^13^. To fulfil this role, learning management skills in midwifery education is necessary^31^. Studies show that midwives recognize the need for systematic education for high levels of management to enable the next generation of midwifery leaders^31^. Midwifery leaders are not only crucial in policy and clinics, but also in research and education^18^.

### Master’s programs in midwifery: Research

Profession-specific research is fundamental for midwifery^18^. Midwives provide their practice based on the best available evidence. The WHO recommends an evidence-based midwifery education^13^. To enable evidence-based education and practice, a wide range of midwifery research is needed^19^. For woman-centered research, midwives themselves should be the researcher. Midwives can understand woman’s needs and the impact of research on them. They understand the practical aspects of midwifery and the reality of clinical research^19^. To become a midwife researcher, there are various options, e.g. Master’s programs in midwifery^19^. They allow midwives to enter a doctoral program, which further trains them as scientific leaders and promotes their academic careers^2^. To pursue an academic career at university, the ability to teach as a midwife is equally important^20^.

### Master’s programs in midwifery: Midwifery educator

Midwifery educators educate midwifery students in theory and practice. This should include midwifery educators who teach in educational settings and clinical midwives who teach in practice, or both^23^. The quality of the educators themselves has a huge impact on the quality of midwifery graduates. It is virtually impossible to train the practitioners, leaders and researchers that are needed if there are not enough well-trained educators in the programs (both Bachelor’s and Master’s programs)^[Bibr cit0020]20^. It is already known that midwifery programs have a shortage of competent midwifery educators^20^. To be a competent midwifery educator, advanced knowledge, skills and behaviors are needed^20^. However, there is a disagreement about what qualifications and competencies midwives need to become midwifery educators^32^. The need for competent midwifery educators is supported by the WHO document ‘WHO Midwifery Educator Core Competencies’^20^. Four of the eight formulated competence domains are at Master’s level^26^. Therefore, the completion of Master’s programs in midwifery can raise the quality of midwifery educators worldwide and therefore improve the quality of care of future midwives^20^. In accordance with the recommendations of the German Council of Science and Humanities, medical faculties can develop their strength here through the combined competencies relevant to the healthcare system^33^. As many Master’s programs in midwifery meet the main areas of leadership and management, research, and midwifery educator^10,21^, some programs go beyond these areas by focusing on a further approach: Advanced midwifery practice (AMP)^22^.

### Advanced midwifery practice

AMP can be defined as: ‘A level of midwifery practice at which midwives use their expertise, management and clinical leadership skills to provide evidence-based, tailored care for woman and their families independently and autonomously. Professional leadership and research skills are used to evaluate practice and advance midwifery as a profession and science’^22^. It is particularly about midwives not only being clinical experts but fulfilling a variety of other roles: consultant, educator, researcher, innovator, clinical and professional leader, policy advisor, and ethical decision facilitator^3,34^. Acquired competencies of AMP extend the areas of leadership and management, research, and midwifery educator by incorporating advanced assessment and intervention strategies, analysis of complex interaction, use of research findings in decision making and management of these, development of woman-centered care and inclusion of education^35^. Studies show that the advanced practice role differs widely from those of clinical specialists. These include satisfaction with service delivery, emotional support and practical advice. Advanced practice roles offer strategic benefits, including better clinical and professional leadership, as well as more research and better service delivery^36^. Education for AMP is structured differently internationally. The entry criteria differ^3,37^, as some expect a Master’s degree for entry to AMP education^35^. Other countries offer AMP education as a Master’s program^38^.

### Rationale of the review

As described above, there are some challenges that cannot be met with a Bachelor’s degree^13^. In fact, a Master’s degree is required for specific challenges that relate to management^13,14,18^, research^19^ and teaching^20,21^ tasks as well as for advanced practitioner roles^22^. As the Master’s programs in midwifery differ both at European^12^ as well as international level^23^, the question arises as to how the Master’s programs address these three areas and how AMP programs go beyond these areas.

To approach this, the review points out the competence goals of the Master’s programs. Competence goals provide a general statement about the expected abilities that graduates of a program should have^39^. To illustrate how the programs address these competence goals, the learning outcomes are examined as well. These make a specific and measurable statement on what a student or graduate will be able to do. A competence goal usually comprises several learning outcomes^39^.

### Objective and research questions

The objective of this narrative review is to provide an overview of Master’s programs in midwifery in selected OECD countries. Besides organizational aspects, the focus lies on competence goals and learning outcomes of the Master’s programs. This provides an overview for the actors involved to develop or enhance Master’s programs in midwifery and to demonstrate the different approaches on how to educate midwives at Master’s level. The research questions were: 1) ‘Which commonalities and differences of organizational aspects in Master’s programs in midwifery occur?’; and 2) ‘On which competence goals and learning outcomes are the Master’s programs in midwifery based on?’.

### The narrative review study design

The study design as a narrative review provides a broad overview of a specific topic as well as quickly inform about the current state-of-the-art^40^. Narrative reviews are considered an important tool in medical education and are commonly used in medical literature syntheses^41^. Since the objective was to obtain an overview of a relevant topic in medical education, this study design was highly suitable. To ensure adequate quality, Scale for the Assessment of Narrative Review Articles (SANRA) was applied^42^. In addition, the Preferred Reporting Items for Systematic Reviews and Meta-Analyses (PRISMA) statement^43^ was considered in the parts suitable for a narrative review (Supplementary file).

*Inclusion and exclusion criteria*

The criteria primarily focused on the Master’s programs. Included were Master’s programs localized at universities or universities of applied sciences in Organization for Economic Cooperation and Development (OECD) countries. This was due to fact that Master’s programs are mostly offered in high-income countries^8^, as well as to ensure that midwives have similar basic conditions for practising as midwives. Still, there are differences in the health systems in every country^44^.

Included were Master’s programs that focused on midwifery science or equivalent as well as advanced midwifery practice. Master’s programs were included that served as pre-service or postgraduate education for midwives. Excluded were Master’s programs that exclusively focused on nursing or related discipline without specific midwifery relation.

### Search strategy and selection procedure

A comprehensive literature search was conducted from June to December 2023. The process was divided into three phases.

*Literature and database search*

In the first phase, the focus lied on a sensitive literature search approach. The aim was to search as comprehensively as possible in different databases with general keywords^45^. Therefore, the following databases were screened: PubMed, ScienceDirect, Educational Resources Information Center, Academic Search Premier and Wiley Online Library. The used search terms were: midwif*, academic education, higher education, master’s degree, master’s program, master*, postgraduate education, advanced practice*, and international*. These search terms were combined in different search strings, which were assembled with the Boolean operators (Supplementary file Tables 1–5). Articles published from the year 2000 onwards and written in English or German were included. In addition, the snowball method was applied (screening the reference list of relevant publications) as well as a free web search on Google to further identify Master’s programs in midwifery.

**Figure 1:**
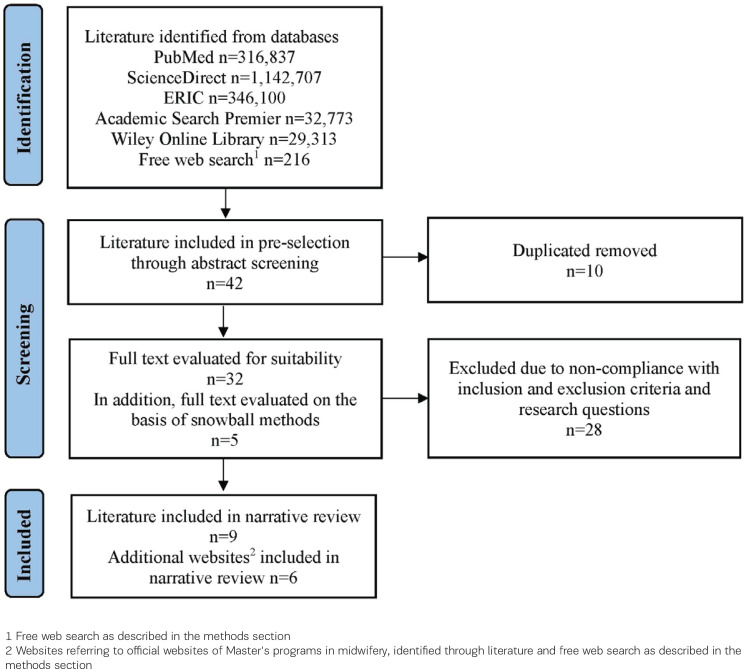
PRISMA flowchart for narrative review (2024)

The search strategy was conducted by two independent researchers. Both researchers documented their search strings and the number of results. After screening relevant articles, each researcher listed their included articles as well as identified Master’s programs. Subsequently, one of the two researchers reviewed the results, which overlapped considerably. This ensured inter-rater reliability^46^. If there were any results that did not correspond, these were verified by a researcher and included if appropriate. The search strategy was documented in a PRISMA flow chart (Figure 1).

*Selection of Master’s programs in midwifery*

After the search for literature, the relevant Master’s programs in midwifery were extracted according to the inclusion and exclusion criteria. These programs were identified by the described process of two independent researchers. The Master’s programs were selected through the identified literature (Figure 1), that referred to institutions of higher education in midwifery. Subsequently, one of the researchers screened the official websites of these institutions for Master’s programs in midwifery. A free web search for Master’s programs in midwifery was conducted by the same researcher in the countries identified through literature search. The described approach to select Master’s programs in midwifery tried to filter relevant programs, as not all OECD countries could be screened in the free web search separately due to insufficient personnel resources. If several relevant study programs were identified in one country, the following procedure was followed: the competence areas and organizational aspects of the study programs were screened. If the programs competence areas differed, both were included. However, if the programs had alike competence areas and differed little in terms of organization, one of the programs was selected at random. This finally led to the selection of Master’s programs in midwifery that met the inclusion and exclusion criteria.

*Identifying the characteristics of the included Master’s programs in midwifery*

In the third and final phase, the included Master’s programs in midwifery were analyzed by one researcher according to the defined educational characteristics. These were synthesized in tabular form for the extracted Master’s programs in midwifery. For the first research question, the following organizational characteristics were extracted: program name, academic degree and institution; entry requirements; course length, full-time or part-time; in person or online; language; tuition; and clinical practice, pre-service education pathway for midwives. Tuition fees were listed in local currencies at the official websites. To ensure comparability, tuition fees have been consistently converted to euros (at the exchange rate on 14th August 2023) and rounded to whole numbers. Focusing on the second research question, the following characteristics were extracted: competence goals and learning outcomes of the Master’s programs. The official websites of the study programs as well as the websites of national midwifery associations were screened for the necessary information.

### Selection of relevant studies

The PRISMA flow chart summarizes the search process (Figure 1). A total of 1867946 results were identified from the databases PubMed, ScienceDirect, ERIC, Academic Search Premier and Wiley Online Library as well as the free web search. In the pre-selection process, 42 abstracts were screened and 10 duplicates removed. After assessing 37 full texts for eligibility, 28 texts were excluded due to non-compliance with the inclusion and exclusion criteria as well as the research questions. Finally, 9 articles and 6 additional websites were included in the narrative review. After reviewing the extracted literature (Figure 1), 12 countries and 15 Master’s programs in midwifery were included in the narrative review (Tables 1 and 2).

**Table 1:** Organizational characteristics of 15 Master’s programs in Midwifery, in 12 OCED countries (2024)

*Country*	*Program name, academic degree and institution*	*Entry requirements*	*Course length full-time or part-time*	*In person or online*	*Language*	*Tuition*	*Clinical practice*	*Pre-service education pathway for midwives*
Australia[57,85]	Master of Midwifery (Research) Faculty of Health, University of Technology Sydney	Applicants must have a recognized Bachelor’s degree or equivalent and be licensed as a registered midwife. Applicants must submit a research proposal and have the approval of an appropriate supervisor. English language skills at an advanced level.	Full-time in 2 years or part-time in 4 years	In person	English	For domestic students, tuition fees are covered by a Research Training Program Scholarship from the Australian government. Tuition fees for international students vary depending on the course at a range from €11825 to €20313 per year.	No clinical practice	There are different ways to obtain the registration as a midwife. These include a Bachelor’s degree in Midwifery, double degree programs combining Bachelor in midwifery with a Bachelor in nursing, and postgraduate programs (e.g. Master’s program). All entry-level programs must meet national accreditation standards for midwifery education[86,87].
Australia[51]	Master of Midwifery Studies The University of Newcastle, Australia	Domestic applicants must be a registered midwife with honors or postgraduate qualifications or at least one year’s professional experience. International applicants must be a registered midwife or nurse working in maternity environment in their country and have at least one year’s professional experience.	Full-time in 1 year (international and domestic students) Part-time in 4 years max (only for Australian students)	Online or in person	English	International students €24425 (full-time) Domestic students €1278 per course (10 units)	No clinical practice	See above
Austria[38]	Master of Science in Advanced Practice Midwifery Health University of Applied Sciences Tyrol	Bachelor’s degree in midwifery, post-secondary midwifery education, comparable Bachelor’s degree programs at a university of applied sciences or university, plus record in selected core subject areas.	Part-time in 2.5 years	Online and in person	German	1st-4th semester €2200 each, 5th semester €700 + €23 per semester for student fees	Recommendation to work in professional practice alongside the study program (but not compulsory)	Midwifery education is a three-year Bachelor’s program at universities of applied sciences that provide both the academic degree of Bachelor and professional licensure as a midwife. Master’s degree is optional for further professional education[88].
Austria[10,49]	Master of Science (Midwifery) University of Applied Science Salzburg, Urstein Campus	Bachelor’s degree in midwifery or graduates of midwifery academy or equivalent state-recognized educational institution in post-secondary sector of at least six semesters plus proof of academic competences, and socialcommunicative skills.	Part-time in 2 years	In person	German, English	€11000 + Student union fee	Supervised clinical reasoning	See above
Germany[55,89]	Master of Science Midwifery and Women’s Health Eberhard Karls University Tübingen	License to work as a midwife, Bachelor’s degree in midwifery science or related field with an average grade better than 2.5, sufficient German language skills.	Full-time in 1.5 years	In person	German and English	Per semester €158 for EU/European Economic Area students Per semester €1500 for international students outside EU/European Economic Area	A mandatory internship can be completed in midwifery facilities, clinics, research institutions or in the field of healthcare management in Germany or abroad	To become a midwife, a Bachelor’s degree of 3–4 years must be completed. After graduation, midwives receive the academic degree Bachelor of Science and the professional license as a midwife. Master’s programs can be completed as a further qualification[90].
Ireland[52,91]	Master of Science in Midwifery Practice and Leadership School of Nursing & Midwifery, Trinity College Dublin, The University of Dublin, Ireland	Applicants are registered as midwives in the Nursing and Midwifery Board of Ireland or as international applicants, registration as a practising midwife in their own country, at least one year of full-time clinical midwifery practice since registration as a midwife, and an honors degree in midwifery or related discipline or equivalent professional and academic qualification.	Full-time in 1 year Part-time in 2 years	In person (combination with blended learning)	English	Year 1 New Student Fee EU €10900, Non-EU €21790	No clinical practice	To become a midwife there are two pathways: an undergraduate honors Bachelor’s degree in midwifery as well as a post-registration through a higher diploma in midwifery for registered general nurses. They both lead to a registration as a midwife in the Nursing and Midwifery Board of Ireland. Master programs can be obtained for further professional qualification or to register as Advanced Midwifery Practice[92].
Ireland[53]	Midwifery-Advanced Practice (AMP), Master of Science/Postgraduate Diploma School of Nursing & Midwifery, Trinity College Dublin, The University of Dublin, Ireland	Degree with honors in midwifery studies or equivalent, current registration of Nursing Division maintained by the Nursing and Midwifery Board of Ireland, at least three years post-registration clinical experience in the last five years, including at least one year of full-time experience in specific area of practice, written commitment from host institution for clinical practicum, host institution verifies clinical audit as suitable learning environment.	Part-time in 2 years	In person (combination with blended learning)	English	Year 1 New Student Fee EU €5792 Non-EU €11792	In the first year, a minimum of 500 hours must be worked in practice under the supervision of an experienced senior clinician	See above
Malta[58]	Master of Science in Midwifery University of Malta (L-Università ta’ Malta), Malta	Bachelor of Science degree (honors) of a university or other higher education institution directly related to the area of study or equivalent; Secure English language test.	Part-time in 3 years	In person	English	Per semester €400 for EU/European Economic Area students Non-EU applicants: total tuition fees €13400	No clinical practice	Midwives are educated based on European standards of higher education for midwives. This requires four years of full-time study to obtain a Bachelor of Science degree and a licence to practise as a midwife. Master’s programs serve as further occupational qualification[61,93].
Netherlands[54,72]	Master of Science Midwifery Midwifery Academy Amsterdam Groningen, Midwifery Academy Maastricht, Midwifery Academy Rotterdam	Registered midwife, Bachelor’s degree in midwifery or equivalent	Part-time in 2 years	Partly online, partly in person, courses are offered in different locations in the Netherlands	Dutch and English	€2128 per academic year for domestic students and EU/European Economic Area students Higher tuition fees for abroad students	Students should work in obstetrics, as the teaching concept provides for practical assignments	A four-year Bachelor’s degree is required to become a midwife. After graduation, a Dutch midwife must register in nationwide health professions register to practice as a midwife. Master’s degree programs can be completed for further professional qualification[94].
Norway[56]	Master in Midwifery Norwegian University of Science and Technology, Trondheim, Norway	Bachelor’s degree in nursing, at least 2 years of professional practice as a nurse, equivalent to a 100% position, on average grade C or better in Bachelor’s degree.	Full-time in 2 years	In person	Norwegian	No tuition fees for students from the EU/European Economic Area students For international students: tuition fees will be announced in the acceptance letter	Even distribution between practical and theoretical courses, practical courses are carried out in primary and specialist health services	Midwifery education is a 2-year specialization after a Bachelor of Science degree in nursing. Graduates qualify for professional license and a Master’s degree in midwifery[95,96].
Portugal[50,97]	Maternal Nursing and Obstetrics Master’s Degree University of Évora, S. João de Deus School of Nursing	Bachelor’s degree in nursing or equivalent, foreign academic higher education in nursing according to the Bologna Process or equivalent, at least two years of professional experience as a nurse.	Full-time in 2 years	In person	Portuguese, English in some curricular units available	€2000 for students from EU countries, €2500 for international students, €2000 for international students with merit scholarships, €2200 for international students with cooperation and development grants	Professional internship with final report	Requirement of four years of nursing education at a university and at least two years of clinical practice as a nurse. After that, a two-year midwifery Master program in a university can be completed, after which graduates receive the academic degree (Master) and can work as a midwife[97,98].
Switzerland[48,99]	Master of Science Midwife Zurich University of Applied Sciences, School of Health Sciences, Institute of Midwifery and Reproductive Health, Winterthur, Switzerland	Bachelor’s degree in midwifery from a university in Switzerland or abroad or midwifery diploma with additional qualifications, good knowledge of scientific work and clinical assessment, sufficient English knowledge.	Full-time three or four semesters (1.5–2 years) Part-time six semesters (3 years)	Online and in person	German, partly English	€750/Semester	Internship at the Institute of Midwifery, teaching or practice area at the University of Zurich, practical internship abroad possible	Bachelor’s degree in midwifery that enables for registration as a midwife as well as obtaining the academic degree Bachelor of Science. Master’s degrees are optional for further specialization of midwives[100].
Türkiye[59,101]	Master’s degree in Midwifery Tokat Gaziosmanpasa University, Faculty of Health Sciences, Türkiye	Bachelor’s degree, additional entrance examination for academic postgraduate programs (must pass with a certain number of points)	Full-time in 2 years	In person	Turkish and English, Turkish level B2 expected	For applicants who have not completed their Bachelor’s degree at that university: Registration Fee: €704 +Each Semester €165 Semester Fees	Internships of the vocational courses under supervision	The undergraduate qualification to become a midwife is based on European and Turkish guidelines of higher education for midwives. Therefore, a 4-year (8 semester) Bachelor’s program enables the qualification of a midwife as well as an academic degree. Master’s programs provide further professional qualification[59,102].
United Kingdom[60]	Enhanced Midwifery Care Master of Science City University of London	All entrants must be qualified midwives; second-class honors degree or equivalent or lower qualification such as Diploma in midwifery with experience, professional experience as a midwife for at least one year; evidence of proficiency in English language or English as first language; for other countries than UK there may be other requirements to enter.	Part-time in 2–3 years Full-time in 1–2 year	In person as well blended learning approaches	English	Full-time students per year: €13750 (for UK students), €16372 (for international students) Part-time students per year: €6875 (for UK students), €8186 (for international students)	Possibility for clinical modules, where students work once a week in a clinical setting under mentorship from a doctor or midwife	To become a midwife, a recognized study program must be completed at a university leading to a registration as a midwife. These take at least three years. In addition, nurses who are already registered can follow an additional program of education that lasts two years and leads to a professional midwifery license. Some Master’s programs allow for midwifery licensure at Master’s level, or as a further professional qualification[103].
United States of America[24,47]	Master’s of Science in Midwifery Thomas Jefferson University, College of Health Professions, Philadelphia, Pennsylvania, United States	Bachelor’s degree from regionally accredited institution or equivalent, licensed as registered nurse in the United States, or live in one of the states where the certified midwife is licensed and complete science and health coursework prior to admission, acceptation only if U.S. citizens and permanent residents.	2 years full-time 3 years part-time	Online and in person	English	For one academic year Full-time: Tuition €39376 + General Fee €1005 Part-time: Tuition €1238€/credit + General Fee €29/credit	Integration of clinical practice and two intensive on-campus courses	All accredited programs for midwifery education require a Bachelor’s degree. Furthermore, it is often necessary to be a registered nurse. There are also some programs with options for those with degrees in other disciplines. Many programs expect a Bachelor’s degree in nursing. The type of midwifery degree may vary from program to program (all award Master’s Degree, Doctor of Nursing Practice (DNP) or Doctor of Midwifery (DM) degree, some offer post-graduate certificate, some offer Master’s completion option those who do not have Master’s degrees)[104].

**Table 2:** Competence goals and learning outcomes of 15 Master’s programs in Midwifery, in 12 OCED countries (2024)

*Country*	*Program name, academic degree and institution*	*Learning outcomes*	*Competence goals*
Australia[57,85]	Master of Midwifery (Research) Faculty of Health, University of Technology Sydney	Learning outcomes of the program are: The practice and demonstration of woman-centered care, taking into account the needs of woman, their babies and families. Appreciate the centrality of the relationship with each woman. Promoting continuity of midwifery care. Creating an optimal birthing environment. Providing effective and safe midwifery care. Solid knowledge of anatomy and physiology. Critical thinking and sound judgement within the framework of current guidelines. Communicate effectively verbally, in writing and non-verbally to different audiences. Utilize information technology. Demonstrate competences in clinical skills. Enable collaboration for excellence in midwifery care. Influence the development of midwifery through leadership, mentoring and role modelling. Work respectfully and collegially as an effective team member. Being a resilient, competent midwife who promotes human wellbeing. Practising and promoting principles of self-care and resilience. Socially responsible to value the diversity of people. Consideration of the philosophy of primary health care. Demonstrate knowledge of the health system. Effective care for woman from diverse backgrounds. Embracing lifelong learning. Interpret and evaluate the evidence that informs practice to influence change. Integrate strategies for personal and professional development. Actively contribute to the development of midwifery. Cultural competence that contributes to the health and wellbeing of Indigenous Australians. Respect and value different worldviews and diversity, particularly of Indigenous Australians, and integrate this into practice.	Key competences within the module Health Care Research Methodology are: Students are able to critically evaluate appropriate methods for a research area as well as integrate the research into existing knowledge. In addition, the ability to write a proposal is addressed, as well as ethical and practical abilities to carry out research in the health sector. Key competence within the module Master of Midwifery (Honors) Thesis is the ability to produce a research thesis.
Australia[51]	Master of Midwifery Studies The University of Newcastle, Australia	Learning outcomes of the program are:Comprehensive knowledge of midwifery studies. Effective written and/or verbal communication skills within an interdisciplinary team. Apply advanced knowledge of midwifery. Comprehensive knowledge of midwifery care of Indigenous woman of childbearing age. Understanding of research methodology and its application to research questions in midwifery and related disciplines. Demonstrate professional self-efficacy and leadership as well as cultural competency and safety. Respect for the community and environment.	Key competences within the module Contemporary Midwifery are: Students are able to demonstrate leadership competences within midwifery profession and maternity care. The ability to identify factors to optimize outcomes as well as to develop tools to measure relevant outcomes for childbearing woman and neonate are demonstrated. Students are able to keep their practice current and evidence-based. Key competences within the module Midwifery and Cultural Diversity are: Students are able to analyze issues related to maternity services for childbearing woman and neonates, considering social determinants of health and human rights, at international and national levels. The ability to critical explore political and cultural context of childbearing in Australia for asylum seekers, refugees or immigrants is enabled as well as to make high-level judgements about maternity care, considering woman’s rights in childbearing and outcomes for woman and neonates. Key competences within the module Optimizing Childbearing are: Students can optimize biological, environmental, social, emotional and psychological factors for woman and babies during the childbearing years. Students are enabled to understand normal psychophysiology and factors that optimize maternal and fetal health from preconception to six weeks postpartum (through abilities in the areas of health and nutrition of both parents, genetics and epigenetics and their effects on woman and their babies, ideal birth environments, interprofessional collaboration, woman-centered midwifery care, birth service design and related policy). Key competences within the module Introduction to Research in Health Care are: Students are enabled to conduct research in health care through different approaches. Abilities in study planning and design are gained. Students have the ability to review and critique published research. Domestic students can take four specializations (midwifery primary care, clinical education; research or leadership and management (offered on part-time basis only), for international students or students who do not wish to specialize, they take general courses.
Austria[38]	Master of Science in Advanced Practice Midwifery Health University of Applied Sciences Tyrol	Learning outcomes of the program are: Advanced professional and methodological knowledge. Embedding this in a scientific, evidence-based context. Leading targeted training units for the optimal medical care of pregnant woman, woman in labor and newborns and preterm births. Being project leaders for the development of emergency strategies. Recognizing psychological stress factors enables intradisciplinary networking. Take on management tasks or teaching activities.	Key competences within the module Obstetric Emergencies and Peripartum Diagnostics are: Abilities relating to obstetric emergency situations and peripartum monitoring are enabled. Knowledge of current obstetric instruments and diagnostic procedures is gained. Key competences within the module Psychosocial Aspects in Obstetrics are: Abilities in psychological and ethical aspects of prenatal diagnostics and intercultural factors in obstetrics are gained. Abilities in relation to the bio-psychosocial anamnesis model, modern pain management and trauma psychology or early recognition of violence are acquired. Key competences within the module ‘Behavioral training modules for management positions in healthcare institutions; personality development; language and communication theory models’ are: Communication competences and abilities in personality development as well as leadership enable leadership positions or project responsibilities. Abilities to analyze communication processes as well as dealing with power conflicts and gained. Students are enabled for tasks in fault management as well as quality management. Key competences within the module Research are: Abilities to use research methods appropriately and to validate and interpret study results. Students can publish scientific papers and reflect on their role as experts in scientific discussions. The program concludes with a Master’s thesis, which provides advanced competences in the field of midwifery research.
Austria[10,49]	Master of Science (Midwifery) University of Applied Science Salzburg, Urstein Campus	Learning outcomes of the program are: Gain advanced knowledge of scientific theories and methods for testing the quality and relevance of evidence. Generating relevant research questions. Have detailed knowledge of pregnancy, birth and motherhood in the field of physiology. Have systematic understanding of physical, emotional and social behavior levels through a salutogenetically oriented consultation and care approach. Gain profound knowledge in the specialization of person-centered communication, counselling and bodywork with the aim of health promotion and prevention. Promote the potential of mother-child, family and society. Have deepened knowledge in self-reflection and reflection of clinical practice for better quality.	Key competences within the module Principles of Salutogenesis are: Advanced theoretical and practical abilities focusing on methods and practices that promote health and health potential. Abilities in counselling and practical guidance towards empowerment and sustainable health-promoting outcomes. Abilities in multidimensional teaching and learning. Key competences within the module Midwifery Research and History of Science are: Abilities to lead an interdisciplinary, professional discourse to improve the quality of obstetric care for healthy pregnant woman / mother-child. Abilities to reflect on and defend arguments and analyses. Abilities for scientific writing and working as well as transfer obstetric knowledge. Key competences within the module Supervised Clinical Reasoning are: Abilities to reflect on clinical practice Key competences within the module ‘Applied salutogenesis, bodywork, communication and didactics’ are: Students acquire social-communicative competences. They gain abilities in the areas of coherence, problem-solving and empowerment. Abilities in didactics, adult education and counselling are acquired. Key competences within the module Current Research are: Abilities to understand key bio-psycho-social processes of adolescence, pregnancy, birth and motherhood. Abilities to understand adaptation mechanisms in their physiological systems. Key competences within the module ‘Public health, perinatal woman’s and family health’ are: Abilities to understand socio-cultural influences on motherhood. Abilities in the field of sociology of woman and body image. Key competences within the module Scientific Methodology are: Abilities for qualitative methods in midwifery research. The program concludes with the completion of a Master’s thesis.
Germany[55,89]	Master of Science Midwifery and Women’s Health Eberhard Karls University Tübingen	Learning outcomes of the program are:Physiological, psychological and social dimensions, system-relevant knowledge and orientation knowledge, aspects of personal development in further education and training, management skills as well as teamwork and cooperation. Dealing with role expectations, personal behavior and the further development of the profession as well as general midwifery theories are enabled.	The program includes 10 modules, which are divided into the four study areas: Management and Leadership, Advanced Midwifery Competence, Women’s Health, and Advanced Research Competence. The modules are aimed at acquiring the following competences, both individually and in interaction with each other: Students acquire advanced personal and academic competences and gain abilities to take on leading positions. Abilities to further develop health care practice are enabled. Advanced scientific and research competences are gained. Students are enabled to define new care paths and extended areas of action for themselves and the profession in an interdisciplinary team. Students gain abilities for education midwives. As an optional module, the qualification to become a practice supervisor can be acquired.
Ireland[52,91]	Master of Science in Midwifery Practice and Leadership School of Nursing & Midwifery, Trinity College Dublin, The University of Dublin, Ireland	Learning outcomes of the program are: To advance midwifery practice and midwifery care through an innovation project in their own practice setting. To investigate and evaluate alternative woman-centered models of midwifery care in Ireland. To explore the origins of midwifery knowledge. To take on leadership roles in healthcare. To promote critical thinking and evidence-based practice. To make their own midwifery research proposal. To reflect on and understand ethical and legal principles for practice. To be able to apply different research methods.	Key competences within the module Advancing the Theoretical Foundations for Midwifery are: Critical reflection abilities in the areas of midwifery knowledge and theory, critical analysis of the relationship between research, theory and practice. Key competences within the module Theory and Practice of Research Methods for Healthcare are: Research and critical thinking abilities to emphasize the importance of best evidence in their area of practice. Abilities are gained to conduct a relevant enquiry into the area of practice. Key competences within the module Ethics and Law in Health and Social Care are: Students gain the abilities to explore legal and ethical knowledge, concepts and frameworks that impact professional practice. Abilities are developed to construct reasoned ethical and legal arguments to proceed appropriately in the provision of health and social care. Key competences within the module Leadership, Quality Improvement and Governance are: To develop abilities appropriate to all roles in clinical practice, speciality nursing and advanced practice to make an effective contribution to the achievement of excellence in patient care. Abilities to develop healthcare policy and service delivery. Key competences within the module Advancing Midwifery Practice are: Abilities for research and understanding the historical and socio-political context of midwifery and obstetrics in Ireland. Key competences within the module Women-Centered Maternity Care in Ireland are: Develop abilities to analyze and reflect on current and changing practice. Develop abilities to critically consider models of care to support the changing healthcare system. Develop abilities to support and develop innovative, woman-centered maternity services. Key competences within the module Dissertation are: Gain abilities to conduct a research study / systematic review.
Ireland[53]	Midwifery-Advanced Practice (AMP), Master of Science /Postgraduate Diploma School of Nursing & Midwifery, Trinity College Dublin, The University of Dublin, Ireland	Learning outcomes of the program are: Pursue a career as an Advanced Midwife Practitioner. To internalize key concepts of person-centered care, autonomy and empowerment, professional ethics, consultation and collaboration, and professional leadership. To practise at the highest professional level throughout the entire spectrum of care.	Competences are gained in the following modules: Professional, Ethical, Legal and Communication Issues informing Nurse and Midwife Prescribing and Clinical Practicum, Decision Making, Midwifery and Advanced Practice Professional roles, Advanced Health Assessment, Prescribing Medicinal Products & Ionizing Radiation, Advanced Practice in the Clinical Practicum with defined pathways Leadership, Quality Improvement and Governance, Advanced Research Methods for Healthcare Professionals, Dissertation in Advanced Practice. [no further information available]
Malta[58]	Master of Science in Midwifery University of Malta (L-Università ta’ Malta), Malta	Learning outcomes of the program are: Learn, develop and apply abilities of scientific enquiry. Conduct a literature search, organize and critique literature. Explore a research question through the selection of an appropriate scientific methodology and data collection method. Develop a research proposal. Conduct research for a Master’s thesis.	The program consists of two modules: Research Methods for Health Care Professionals and Dissertation. Advanced research skills are developed during this program. The abilities to conduct a research project are developed. Abilities to empirically investigate aspects of practice to expand understanding and knowledge in the field are gained.
Netherlands[54,72]	Master of Science Midwifery Midwifery Academy Amsterdam Groningen, Midwifery Academy Maastricht, Midwifery Academy Rotterdam	Learning outcomes of the program are: Take on tasks in the field of leadership. Bridge the gap between the medical and social fields and between day-to-day practice, research and policy. Implement evidence-based findings in practice. Being able to implement innovation.	Key competences within the module Advanced Midwifery are: Abilities to develop professional clinical leadership in obstetrics are gained. Abilities to make critical decisions are acquired, taking into account the concept of salutogenesis. The ability to reflect is developed. The ability to take a clear and well-founded position is acquired. Key competences within the module Leadership, Quality and Policy are: Abilities to consider different perspectives on quality and policy. Abilities to develop policies from a quality improvement perspective. Abilities to fulfil a leadership role. Key competences within the module Research Skills are: Abilities to further develop professional practice. Basic research competences are acquired that are appropriate to the diversity of research questions in professional practice. Advanced abilities are gained in optional modules: Social Midwifery or Organization, Management and Entrepreneurship or Innovation and Implementation. The program concludes with the completion of the Master thesis.
Norway[56]	Master in Midwifery Norwegian University of Science and Technology, Trondheim, Norway	Learning outcomes of the program are: Provide midwifery care at various levels of the healthcare system and collaborate with other stakeholders. Provide expert care to woman during pregnancy, labor and postnatal care and care for newborn babies. To promote the reproductive and sexual health of woman at different phases of their lives. To contribute to the development of research-based midwifery and to communicate and disseminate professional midwifery topics and research findings. Apply scientific and ethical theories and concepts to the development and writing of a Master’s thesis.	The modules Midwifery 1 and 2 are compulsory as well as an Introduction to Research Methods. Optional modules can be chosen between qualitative methods and statistics in medicine and health sciences. The program concludes with the completion of the Master’s thesis. The modules contribute to the development of competences in the following areas: Abilities to perform midwifery tasks at different levels of the healthcare and private sector and to participate in and lead the development of the midwifery profession. Abilities to work as a midwife and clinical management. Abilities for education of midwives and other health personnel, as well as leading positions. Abilities for research and teaching.
Portugal[50,97]	Maternal Nursing and Obstetrics Master’s Degree University of Évora, S. João de Deus School of Nursing	Learning outcomes of the program are: Understand the significance of biological aspects of human development that arise during embryonic development. Acquire in-depth knowledge of human reproductive and development systems. Take care of woman in different stages (periconceptional, conceptional, puerperal) during health deviation, pregnancy interruption or abortion. Identify and monitor high-risk pregnancies and aspects of obstetric pathology. Take care of woman inserted in families during the preconceptual phase. Have nursing knowledge of the prenatal period. Conduct scientific research process in the field of obstetric maternal health. Have evidence-based knowledge for practice and ethics, taking into account human rights and professional responsibility. Recognize the importance of development and trends in obstetric and maternal health care. Know quality programs to develop strategic initiatives. Take care of woman and woman in labor, birth and the postpartum period. Have knowledge of the psychology of pregnancy, childbirth and the postpartum period and the processes of transition to parenthood in health and risk situations. Prepare for childbirth and parenthood. Have socioanthropological knowledge of family realities in today’s society. Reflect on thematic debates about the discussion of family in contemporary society. Understand the importance of breastfeeding and act in accordance with WHO/UNICEF guidelines for the promotion of breastfeeding. To base the theoretical and practical aspects of specialized care on scientific evidence and to reflect critically on practice OR To apply and defend a scientific thesis OR To apply the acquired knowledge in a project using scientific evidence.	Advanced knowledge and skills are gained with the overall competence goal to enable students to take care of woman, inserted in the family / community, during pregnancy-puerperal cycle and take care of sexual and reproductive health. This is enabled through the following modules: Biology of Pregnancy and Motherhood, Anatomophysiology Pregnancy and Puerperal, Obstetrics, Nursing Care at Preconception & Pregnancy, Nursing Research, Evolution and Trends in Obstetrics and Maternal Health Care, Nursing Care at Childbirth and Puerperium, Postconceptional Nursing Care, Preparation for Childbirth and Parenting, Sociology and Anthropology of the Family, Breastfeeding, Professional Internship with Final Report OR Dissertation of Scientific Nature OR Project Work.
Switzerland[48,99]	Master of Science Midwife Zurich University of Applied Sciences, School of Health Sciences, Institute of Midwifery and Reproductive Health, Winterthur, Switzerland	Learning outcomes of the program are: Take on tasks in the areas of specific clinical practice, modern leadership and applied research. Take on the following tasks in the Advanced Midwifery Practice: Clarify needs of woman and families in special situations, develop new models of care and promote continuity of care. Take on a leadership role and support interprofessional teams. Develop care pathways and initiate case reviews and implement new guidelines. Take on the following tasks in research: Elaborate midwifery-specific questions in scientific projects, create the basis for further development of needsorientated care and to make this knowledge accessible to practitioners. Take on the following tasks in teaching: Pass on evidence-based knowledge to students as a lecturer and provide further qualifications. Take on the following task in an academic career: Start a doctorate.	The study program focuses on four module groups: Practice Fields and Competencies, Advanced Practice, Research and Master’s Thesis. Key competences within the module group Practice Fields and Competencies are: Complex obstetric situations: Abilities to analyze complex care situations from self-learned practice and to derive appropriate actions. Global Health: Ability to deal with situations of woman and families with different backgrounds and access to health care with a focus on health equity. Midwife-led obstetrics: Ability to comprehensively consider the processes of pregnancy, birth and parenthood. Antenatal care and support: Ability to identify and analyze national and international models of antenatal care. Key competences within the module group Advanced Practice are: Ability to understand the concepts, roles and tasks of advanced practice. Ability to deal with national and international health policy and related role development. Abilities in communication and coordination techniques and interprofessional communication skills. Key competences within the module groups Research and Master’s Thesis are: Abilities in the areas of scientific theory and methods as well as abilities for quantitative and qualitative research. Ability to write a Master’s thesis, taking the principles of project management and research ethics into consideration. Optional modules are either Advanced Practice Training, Advanced Practice Ethics Workshop or an international internship.
Türkiye[59,101]	Master’s degree in Midwifery Tokat Gaziosmanpasa University, Faculty of Health Sciences, Türkiye	Learning outcomes of the program are: Define the historical development of midwifery and the current situation, basic concepts of midwifery, duties of midwifery, powers and responsibilities in terms of midwifery philosophy. Have basic knowledge of midwifery care and practice as well as about childbirth, woman’s health, family planning, newborn, public health, social sciences and ethics. Have basic medical and scientific information based on midwifery care. Develop midwifery practice through the use of midwifery research and their application in midwifery practice. Follow the activities of national and international professional organizations and to collaborate with professional organizations at national and international level. Plan an action plan in relation to professional problems. Carry out scientific studies in the field of midwifery. Assume a professional identity and role model function.	The study program consists of at least seven courses, other learning activities, thesis work, seminar and specialization courses. Key competences of the study program are as following: Abilities to transform midwifery knowledge into expertise, have comprehensive knowledge and skills, think creatively and critically and combine scientific knowledge with international professional ethics. Abilities to be sensitive to health problems and care needs of individuals, family and society at national and international level. Abilities to solve health problems in midwifery. Abilities to obtain and analyze midwifery information. Abilities to conduct research. Abilities to apply teaching principles and methods in the nursing area. Abilities to carry out independent research in order to apply the results to the benefit of humanity. Abilities to undertake management and administrative tasks in midwifery. Abilities to recognize health needs of individuals, families and society and to carry out midwifery initiatives in any setting. Abilities to adapt and plan midwifery care to meet identified needs. Abilities to recognize health-related educational needs of patients in care and to develop and evaluate an educational plan. Abilities to provide treatment as specified in writing, except in emergencies. Abilities to perform duties as specified in regulations.
United Kingdom[60]	Enhanced Midwifery Care Master of Science City University of London	Learning outcomes of the program are: Promote personal and professional development. Critically reflect on their own practice. Share best practice and clinical experiences across a range of maternity services. Have the critical appraisal and analytical skills for the interpretation and evaluation of midwifery practice. Provide evidence-based midwifery care. Develop independent lifelong learning. Develop expertise. To ignite and inspire passion for midwifery care. Build professional confidence, resilience and innovative thinking.	The program consists of core modules, discipline specific modules and elective modules as well as the dissertation. Key competences of the core module Introduction to Research Methods and Applied Data Analysis are: Develop theoretical, methodological and research abilities. Abilities to conduct research with analytical conclusions as a basis for effective service delivery. Key competences of the discipline specific module Midwifery Theory: Knowledge, Profession and Practice are: Abilities to explore midwifery theories and practice using academic theoretical perspectives. Abilities to explore current challenges and opportunities in organization offering midwifery care. Key competences of the discipline specific module Risk and Midwifery Practice are: Abilities to critically evaluate clinical practice management as well as interprofessional practices. Abilities to critically examine care. Key competences of the discipline specific module Optimum Birth are: Abilities to support physiological birthing process for all women. Key competences of the discipline specific module Public Health, Pregnancy and Parenthood are: Abilities to explore maternal and child health issues and explore the social, political and policy context of health services in the UK. Elective Modules are as following, each supporting different competences: Critical Approaches to Advanced Practice, Advancing Maternity Practice: Facilitating Group Antenatal Care, Advance Physical Assessment of the Newborn and Infant, Health Policy in Britain, Advanced Data Analysis, Cervical Cytology, and Perinatal Mental Health. The program concludes with the dissertation, where abilities for research, data collection, analyses and interpretation are gained.
United States of America[24,47]	Master’s of Science in Midwifery Thomas Jefferson University, College of Health Professions, Philadelphia, Pennsylvania, United States	Students will be eligible after graduation to take the American Midwifery Certification Board national examination and earn the title of CNM or CM. Learning outcomes of the program are: Graduates will be able to internalize the science of midwifery through various forms of teaching and learn the art of midwifery through practice during clinical placements under the supervision of a qualified midwife. Graduates will demonstrate practical skills, clinical reasoning and clinical reflection.	Competences in the following modules are gained: Advanced Physiology/Pathophysiology for Primary Care; Professional Issues; Theoretical Foundations Advanced Health Assessment, Reproductive & Sexual Healthcare, Advanced Pharmacology I, Evidence-Based Care: Evaluating Research, On Campus Intensive: Office skills. Embryology and Genetics, Preparation for Office Based Practice, Antepartum Care, Postpartum/Newborn Care, Clinical Midwifery - Ambulatory Care I. Introduction to Health Policy Intrapartum Care, Advanced Pharmacology II, Clinical Midwifery - Ambulatory Care II, On Campus Intensive: Birth skills, Preparation for Full Scope Midwifery Practice, Clinical Full Scope Midwifery Care I, Advanced Perinatal Pathophysiology, Elective Clinical Full Scope Midwifery Care II, Midwifery Nexus Project

## MASTER’S PROGRAMS IN MIDWIFERY

### Organizational aspects of the reviewed Master’s programs in midwifery

The review included 15 Master’s programs (Table 1) in midwifery from 12 OECD countries: Australia, Austria, Germany, Ireland, Malta, Netherlands, Norway, Portugal, Switzerland, Türkiye, UK, and USA. Australia, Austria and Ireland each include two Master’s programs. Similarities and differences within organizational characteristics of the Master’s programs are identified: all 15 Master’s programs award the title Master of Science^38,47-60^; 13 of the programs focus on Master in Midwifery (or equivalent)^47-52,54-60^, while two of them pursue AMP^38,53^. Six of the programs require a minimum knowledge of English^48-50,54,55,59^. Seven of the programs teach in English^47,51-53,57,58,60^, which is their official language or mother tongue. Eight programs teach in their native language other than English^38,48-50,54-56,59^. In addition, seven of the programs offer their courses both online and in person^47-49,52-54,60^. Major differences between the programs are the admission requirements and qualification. Three of the analyzed Master’s programs are pre-service education for midwives, requiring a Bachelor’s degree in nursing as well as practical experience for admission. These post-nursing Master’s programs allow a first-time registration as a midwife after completion^47,50,56^. 12 of the Master’s programs serve as postgraduate midwifery education. They require a Bachelor’s degree in midwifery and a registration as a midwife for admission^38,48,49,51-55,57-60^. Five of the study programs integrate clinical practice^47,50,53,56,59^, while four allow projects or internships in different intuitions^48,49,55,60^.

Two of the programs recommend working in clinical practice alongside the study course^38,54^, whereas four of the programs do not require any clinical practice at all^51,52,57,58^. Differences are identified in the study length, as durations of 1–4 years in part-time or full-time in six programs^38,47,51,52,57,60^, only part-time with 2–3 years in five programs^38,49,53,54,58^, or only full-time with 1.5–2 years in four programs^50,55,56,59^ are possible. Another central difference is found in the tuition fees that differed enormously between the countries, e.g. more than €40000 per academic year for domestic students in the USA^47^, up to no fees at all for domestic students in Norway^56^.

### Competence goals and learning outcomes of the Master’s programs in midwifery

Similarities and differences can be identified in terms of the competence goals and learning outcomes of the Master’s programs (Table 2). The ability to undertake research is a central competence goal in all 12 programs^38,47-60^. This is accomplished in the different study programs through various learning outcomes. Graduates should be able to apply research methods after completing their studies^48,51,53,57-60^ or have the ability to integrate professional knowledge into a scientific, evidence-based context^38,48,50,54,59^ as well as to promote evidence-based practice^38,50-52,54^. Furthermore, graduates should be able to verify evidence and conduct one’s own research project^38,49-52,57-60^. In this context, seven of the programs educate for the ability to think and reflect critically^47,51,52,55,57,59,60^. Six of the programs also integrate scientific and midwifery theories in their studies^48,49,52,55,56,60^. Ultimately, all these competence goals and learning outcomes in the area of research, aim to enable their graduates to further develop midwifery practice^38,47-60^.

Twelve of the programs focus on abilities for leadership and management^38,[Bibr cit0048]48,49,51-57,59,60^. This is addressed by the learning outcomes of graduates being able for effective teamwork^48,51,55-57^ as well as effective communication^38,48,49,51,53,55-57^. Graduates should be able to take on (clinical) management tasks^38,48,49,52-56,59,60^, such as quality management^38,49,53-55,60^ and/or project responsibilities^38,48,52,53,55^. Moreover, graduates should be able to take on leading positions^38,48,49,52-56^.

A central key competence in seven programs is to enable graduates to educate midwives^38,48,49,51,55,56,59^ in theory and practice^38,48,55,56,59^; for example, in clinical education^51,55,56^ but also for further professional qualifications^48^. In addition, the ability to educate patients based to their informational needs is enabled^59^. To address the mentioned abilities for education midwives, graduates should be able to apply theories of teaching as well as theories of learning^48,49,55,59^.

Another competence goal that can be found in nine of the Master’s programs are abilities for ethical aspects in midwifery and healthcare^38,48,50,52,53,55-57,59^, whereas only four programs enable graduates skills for legal and judicial aspects in midwifery and healthcare^52,53,55,59^. Moreover, eight of the programs focus on the competence goal of abilities for psychological as well as sociological dimensions of midwifery care^38,49-51,54,55,59,60^. A further competence goal of eight programs is to enable graduates’ advanced clinical skills^47-49,52-54,57,60^. Whereas ten programs focus on woman-centered care^38,48-52,55,57,59,60^, five programs focus on family-centered care^48-50,57,59^. Another competence goals in three of the programs is to enable skills in cultural competence^38,51,57^. Competence goals that can only be identified in Master’s programs focusing on AMP are abilities for obstetric emergency situations and peripartum monitoring^38^, trauma psychology^38^, fault management^38^, dealing with power conflicts^38^, prescribing medicinal products and ionizing radiation^53^, as well as abilities to practice with the highest professional level throughout the entire spectrum of care^53^. In general, overlapping competence goals can be identified, whereas the learning outcomes differ to address these competence goals.

### Summary of the findings and answers to the research questions

The narrative review includes 15 Master’s programs in midwifery from 12 OECD countries (Australia, Austria, Germany, Ireland, Malta, Netherlands, Norway, Portugal, Switzerland, Türkiye, UK, and USA). Considering the organizational aspects of Master’s programs in midwifery (answering research question 1), several similarities can be identified. The awarded title Master of Science in midwifery (or equivalent)^38,47-60^ is common to all of the programs, although two especially focus on AMP^38,53^. Basic knowledge in English is required in 13 of the programs^47-55,57-60^. Differences can be identified, among other things, within admission requirements and qualification level (primary qualification^47,50,56^ or postgraduate program^38,48,49,51-55,57-60^) and the integration of clinical practice (from no clinical practice^51,52,57,61^ to integrating clinical practice or projects^38,47,48,50,53,55,56,59,60^).

Analyzing the competence goals and learning outcomes of the Master’s programs (answering research question 2), commonalities can be found within the competence goals. The objective for research competence underlies all 15 programs, with the overall purpose of further developing midwifery practice^38,47-60^. Another competence goal of 12 the programs is the ability for leadership and management^38,48,49,51-57,59,60^, which is mostly addressed by graduates being able for effective teamwork^48,51,55-57^ and effective communication^38,48,49,51,53,55-57^. Furthermore, seven of the programs aim for the ability to take over tasks in midwifery education^38,48,49,51,55,56,59^. Other competence goals are the ability to deal with ethical aspects of midwifery and healthcare^38,48,50,52,53,55-57,59^, as well as psychological and sociological skills^38,49-51,54,55,59,60^. In addition, graduates are enabled for advanced clinical skills^47-49,52-54,57,60^, mostly focusing on woman-centered care^38,48-52,55,57,59,60^. Although the overarching competence goals mostly align, differences can be identified within the learning outcomes to address these competences. For example, while five of the programs educate research competence by graduates being able to promote on evidence-based practice^38,50-52,54^, 10 of the programs focus on graduates being able to implement a research project^38,49-52,57-60^.

### Competences of midwives with a Master’s degree: Management and leadership

The SoWMy states that investment in midwifery leadership and governance are urgently needed to improve quality and safety of maternity care^8^. To enable strong leadership, the roots are already planted in the education of midwives^18^. The integration of leadership and management skills is recommended for undergraduate and postgraduate education^62^. However, many midwives are not able to find appropriate leadership and management positions after their primary qualification due to a lack of leadership opportunities^8^ or not having enough specialized leadership skills^63^. In addition to professional work experience^64^, further education that develops specific management and leadership skills is useful^65^. This is reflected within the analyzed Master’s programs, as many of them focus on abilities for management and leadership^38,48,49,51-57,59,60^. To fulfil management and leadership tasks, midwives should have knowledge about cooperate management issues to integrate and understand policy direction and commissioning. Moreover, midwives in leadership and management positions should have the ability to understand the wider healthcare system. Midwives need to develop negotiation, persuasion and influencing skills as well as political astuteness to take on leadership and management roles. To build these competences, midwives need to understand the structure as well as the language of the systems they are working in, especially focusing on safety and quality, finances and resources as well as workforce planning and modernization^66^. In general, these competences are found in the Master’s programs examined as their learning outcomes focus on graduates being able to take on clinical management tasks^38,48,49,52-56,59,60^, but it is not clear within the scope of the review to what extent these abilities are developed.

In addition to contextual understanding, professional experience is equally important. This includes, for instance, experience in project management^64^, which is also a central learning outcome of many Master’s programs examined^38,48,52,53,55^. Furthermore, the ability to communicate effectively is a central component of midwifery leadership. Another central ability is to lead teams towards visions and goals they share^64^. Both effective teamwork^48,51,55-57^ as well as effective communication^38,48,49,51,53,55-57^ is trained within the Master’s programs. After all, midwifery leadership and management is about changing the future of midwifery^64^, which many of the analyzed Master’s programs refer to^38,47-60^.

### Competences of midwives with a Master’s degree: Midwifery research

Research is an integral part of midwife’s role^19^. Internationally, research opportunities and careers in research for midwives are increasingly expanding. This is necessary to provide efficient and cost-effective care that adapts to changing needs of consumers and society^67^. In its ‘International Code of Ethics for Midwives’, the ICM describes that midwives use up-to-date data and professional evidence-based knowledge for safe midwifery practice as well as actively participate in research^68^. However, evidence-based practice and research are seen as a challenge for midwifery students^69^ as well as practitioners^21^. A tension between evidence-based practice as well as research and practice is reported^69^. Reasons for this include the lack of clinically active midwives with solid scientific training in many places have^67^ and their lack of influence on the framework conditions and the control of these via management positions^8^. Embedding elements of evidence-based practice and research in the primary qualification of midwives is not enough to ensure that evidence-based practice can be implemented within the actual work as a midwife^69-71^. Rather, to prepare midwives for research activities, they should complete Master’s or doctoral programs^19,67^, where comprehensive research abilities are developed. A highly relevant ability for midwifery research is the ability for interdisciplinary cooperation. The reason lies within the fact that the contribution from other disciplines and interdisciplinary exchange are seen as extremely valuable source of knowledge to midwifery research^72^. This is enabled by most of the analyzed Master’s programs by the learning outcomes of graduates being able for effective teamwork^48,51,55-57^ as well as for effective communication^38,48,49,51,53,55-57^. However, despite the relevant contribution of other disciplines, it is necessary to find a separate identity for midwifery research with a perspective on midwifery care. Therefore, midwifery researchers should be familiar with appropriate qualitative and quantitative methods and balance these approaches carefully^72^. The analyzed Master’s programs address this by the learning outcome of graduates being able to apply different research methods^48,51,53,57-60^. Despite the abilities to conduct research^72^, transferring findings into evidence-based practice is an essential ability for midwives. Studies show that this is enabled by reading and reviewing literature as well as translating these findings into evidence-based practice^19,73^. Moreover, midwives should be able to disseminate literature to health professionals in a purposeful way^73^. Many of the Master’s programs analyzed here focus on the named abilities for transferring findings into evidence-based practice^38,48,50-52,54,59^. After all, midwifery research refers to how midwives work and how midwifery practice can be improved^72^, which is why qualified midwives with a Master’s degree are needed^19^.

### Competences of midwives with a Master’s degree: Midwifery educators

To educate competent midwives and enable midwives for careers in research, leadership and practice, it is essential to have competent midwifery teachers^20,74^. Of the 15 analyzed Master’s programs in this review, only seven explicitly refer to the competence goal of midwifery educator^38,48,49,51,55,56,59^. However, when considering the midwifery educator competency domains defined by the WHO^20^, many of the programs prepare graduates for those competency domains, even without making explicit reference to midwifery educator. According to the WHO, midwifery educators should base their knowledge and skills of midwifery on the best available evidence^20^. All analyzed programs refer to the competence goal of research abilities^38,47-60^ as well as to the learning outcomes of graduates being able to promote evidence-based practice^38,50-52,54^. Another research-related competency domain of the WHO refers to midwifery education and practice being informed and developed through research^20^. All the analyzed programs aim to enable graduates to further develop midwifery practice through research competence^38,47-60^. In addition, another competency domain of the WHO focusses on effective communication, acting as advocates, change agents, and leaders^20^. This is also reflected in many of the considered programs, as they enable graduates for leaders^38,48,49,51-57,59,60^, as well as for effective communication^38,48,49,51,53,55-57^. However, there are other competency domains of the WHO, referring to pedagogical and didactical skills, such as creating an environment for facilitating teaching and learning^20^. These areas are not met within many of the analyzed programs, as only a few train abilities for teaching and learning^48,49,55,59^. This can be seen critically, as adult education requires specialized teaching skills. These include knowledge in adult education theories, the ability to apply teaching methods effectively, and therefore support students and their individually needs^74^.

### Competences of midwives with a Master’s degree: AMP profile

As some of the analyzed Master’s programs qualified for AMP, the question arises to what distinguishes them from the other programs examined. The competence areas of leadership and management, research and education are also represented within the AMP Master’s programs. However, AMP programs emphasize an explicitly focus on leadership and research^38,53^. Literature shows that research skills are an essential attribute of AMP, which is why AMP Master’s programs place major focus on this competence^22^. Moreover, leadership skills of advanced practitioners are highlighted as an increasingly important factor for improving patient outcomes. Although, as already argued, all midwives with a Master’s degree should be suitable for management positions, advanced practitioners are particularly well-suited as have additional responsibility for professional development and a high level of expertise. Moreover, they have complex decision-making skills^73,75^. That is also reflected within the analyzed AMP Master’s programs as they specialize on the entire spectrum of care by educating clinical decision-making as well as emergency strategies^38,53^. AMP programs develop skills that enable a higher level of judgement, discretion and decision-making in all practical settings^37^. They also educate for specific diagnostic skills^37^, which can be found in one of the analyzed Master’s programs as competence goals^38^. In conclusion, AMP Master’s programs extend the other programs by enabling specialization and expansion of knowledge, abilities and autonomy in the named areas and beyond^22^.

### Post-nursing and postgraduate Master’s programs in midwifery

There were two different types of Master’s programs included in this review (post-nursing and postgraduate Master’s programs in midwifery). On the one hand, it is known from related disciplines that postgraduate Master’s programs improve knowledge and skills as well as improve patient outcomes^76^. With postgraduate Master’s programs midwives have further career opportunities, such as in research, education, management or consultancy. Moreover, besides personal benefits for graduates, Master’s programs in midwifery provide the necessary skills to maintain the autonomous professional role of midwives^77^. Studies also show that midwives have a higher level of pedagogical, management and research skills after completing a postgraduate Master’s program. However, there is still a lack of opportunities to apply these skills in practice^21^. On the other hand, it is known from nursing that a four-year primary qualification is associated with better performance and patient outcomes^78^. The post-nursing qualifications for midwives usually have a longer duration. As a result, a high amount of flexibility of graduates is reported^79^. Studies indicate that graduates of post-nursing Master’s programs benefit from changes in attitudes, perceptions, knowledge and skills as well as their application in practice^80^. Overall, there is very little evidence on how competencies, costs, career paths or other outcomes differ from each other considering the different educational pathways of midwifery^9^.

### Adaption of Master’s programs to country-specific characteristics

While there are many standards for undergraduate midwifery education^14-16^, much fewer standards exist for postgraduate education as they only refer to general classifications of education^28,29^. There were clear patterns in terms of competence goals and learning outcomes of the analyzed Master’s programs that could also be embedded in literature (see above). Nevertheless, is should be noted, that country-specific adaptions for postgraduate education are necessary. The reason lies within the fact that there are considerable differences in the integration of the midwifery profession into healthcare systems, both within and between countries^8,44^. This ranges from regulation and scope of practice to involvement in care^8,44^ as well as different educational systems^8,9^. The influence of the professional organizations on the curricula and the different educational programs also varies from country to country^81^. Considering the analyzed competence areas, it is clear that midwifery research is a country-specific phenomenon, as each country approaches midwifery research differently, especially in terms of addressed topics and integration of research in the professional role for midwives^72^. As the career opportunities for midwifery researcher differ internationally^19,72,82^, the country-specific characteristics of midwifery research is needed to be considered in the various Master’s programs. Considering the competences of midwifery educators discussed^20,26^, some of them are specifically aligned towards national healthcare systems. For example, midwifery educators should be able to understand and demonstrate the ethical and legal principles of midwifery^20^. Whereas the ethical principles of midwifery are recognized in official international documents of the ICM^68^, there are country-specific characteristics especially in terms of regulatory and legal aspects of midwifery^8^. For effective midwifery leadership, the ICM recommends focusing on the structures of each country and its specific healthcare system^18^. Consequently, it is clear, that a systematic adaption of the Master’s programs and their competence goals to country-specific characteristics of their healthcare systems^8,21^, their integration of the role of midwives^21^, as well as educational systems^8,9,81^, is considered reasonable and necessary.

### Recommendations for research

Similarities and differences of organizational aspects as well as of competence goals and learning outcomes were identified. Three research gaps emerge from this review: 1) The analyzed similarities and differences can serve as an indication for further research as Master’s programs of many countries need to be analyzed in detail to collect more data. The purpose of further research is to find the core elements of Master’s programs in midwifery that are equally important for each program; and 2) This review has also shown that in addition to these core elements, there are relevant country-specific characteristics each Master’s program has to address differently. To fully understand which organizational aspects as well as competence goals and learning outcomes need to be adapted to individual circumstances, further research is necessary; and 3) Finally, it is important to examine the impact of Master’s programs, especially regarding the educational pathways, on health outcomes and national healthcare systems.

### Limitations

A central limitation of this review is the study design. The narrative review follows a systematic approach^42,43^, nevertheless, it is possible that relevant Master’s programs and publications have been missed. In conducting the search for identifying Master’s programs in midwifery, the primary source of information was academic literature. A free web search was consulted only secondarily. Consequently, it can be assumed that a number of relevant programs were overlooked. Some countries, e.g. Greece, have a number Master’s programs in midwifery^83^ that were not identified through the aforementioned method. Moreover, the needed information on the characteristics of the Master’s programs is mostly found on the official websites of their institutions, rarely in scientific articles. Still, it can be assumed that the universities have trustworthy websites. As the search was mainly conducted in English, and partly German, study programs which maintain their websites only in their native language (if not English or German) and do not have corresponding publications, could not be identified. Another limitation is the different health systems and midwifery roles as the Master’s programs originate in different countries^44^. By focusing on OECD countries, up to a certain point a comparison is possible, nevertheless significant differences exist in the health and education systems even within these countries^84^. However, this can also be seen as being positive, as the countries can learn from each other. Beyond these limitations, the study did perform a systematic approach to the literature search (as far as possible). Two independent researchers conducted the literature search, and each listed separately their search strings, number of results, relevant articles and Master’s programs. The results overlapped considerably, which increases the inter-rater reliability^46^. The results were, however, analyzed by one researcher only. Overall, the generalizability of the results is limited by the named aspects. Still, the results allow a solid overview and provide indications for practitioners, researchers and other involved actors.

## CONCLUSION

Master’s programs in midwifery prepare for challenges that cannot be met with a Bachelor’s degree^13^. These especially refer to management^13,14,18^, research^19^, teaching^20,21^ tasks as well as advanced practitioner roles^22^. This review analysed 15 Master’s programs in midwifery in 12 selected OECD countries. Organizational commonalities (e.g. awarded title) as well as organizational differences (e.g. admission requirements) were identified. There are central core elements that underlie most of the Master’s programs analyzed, especially referring to competence goals. Nevertheless, these competence goals are addressed differently through adverse learning outcomes. As there is a need to adapt the Master’s programs to the national context, further research on core elements as well as adaption to country-specific characteristics is needed^8,9,21,81^. As there is little evidence on Master’s programs in midwifery, this review serves as a starting point for further research.

## Supplementary Material



## Data Availability

Data sharing is not applicable to this article as no new data were created.
